# Supplementary material for: Effects of Dietary Fiber Sources during Gestation on Stress Status, Abnormal Behaviors and Reproductive Performance of Sows
Source: Animals (Basel). 2020 Jan 15;10(1):141. doi: 10.3390/ani10010141 (PMC7022560; doi:10.3390/ani10010141)
Supplement: Supplementary file 1 [file animals-10-00141-s001.pdf]

animals

Effects of Dietary Fiber Sources during Gestation on Stress Status, Abnormal Behaviors and Reproductive Performance of Sows

Shuangbo Huang [1], Jianfu Wei [2], Haoyuan Yu [1], Xiangyu Hao [1], Jianjun Zuo [1], Chengquan Tan [1,3,*] and Jinping Deng [1,*]

Table S1. Behavior and definition.

**Behavior**	**Definition**
Drinking	Manipulating the nipple drinker
Sham-chewing	The behavior of the sow's mouth in the absence of food
Sniffing, licking	Nasal stabilization or tongue contact with the ground, railings, or no water trough
Standing	Body supported by four legs, with or without stamping
Sitting	Body supported by the front legs, with hind legs and bottom flat on the ground
Lying	Body supported by less than three legs
Position change	Any motion involved in the transition of postures

Table S2: The number of sows during the experimental period

Item	Diet		
	**CON**[1]	**RS**[1]	**FSF**[1]
Mating	26	26	26
At day 109 of gestation[2]	22	21	24
Farrowing	22	21	24
Culled during lactation[3]	3	0	2
Weaning	19	21	22

[1] CON—control diet group; RS—5% resistant starch diet group; FSF—5% fermented soybean fiber diet group; [2] Sows were culled because of foot pain, abortion, or non-pregnant after breeding, etc.; [3] Sows were culled because of poor milk.
